# Seismic Risk of Critical Transport Systems: Sensitivity Analysis of the Campania Railway Network to Bradyseism's Features

**DOI:** 10.1111/risa.70326

**Published:** 2026-08-06

**Authors:** Raffaele De Risi, Francesco Pugliese, Jitendra Agarwal, Francisco Pinto

**Affiliations:** ^1^ School of Civil, Aerospace and Design Engineering University of Bristol Bristol UK; ^2^ School of Civil Engineering University of Leeds Leeds UK; ^3^ Departamento de Ingeniería Civil Universidad de Chile Santiago Chile

**Keywords:** epistemic uncertainty, global sensitivity analysis, ground‐motion model (GMMs), railway infrastructure risk, volcanic seismic hazard

## Abstract

This study examines the impact of epistemic uncertainties on the seismic risk of a critical transport system, the Campania railway network, during the ongoing bradyseismic unrest at Campi Flegrei, south of Italy. Seismic risk is quantified as the length of the railway requiring post‐event inspection, an operational proxy for service disruption. A scenario‐based risk formulation is adopted, integrating fragility functions for tunnels and masonry arch bridges with a ground‐motion model tailored explicitly to volcanic areas. Peak ground acceleration serves as the intensity measure, whereas the annual acceptable probability of failure from the Eurocode informs safety decisions. Epistemic inputs comprise epicenter‐location error and azimuth, duration–magnitude error, central‐value variability in the ground‐motion model, a soil term, and median fragility parameters. A regional sensitivity analysis (SA) using Latin hypercube sampling reveals that uncertainties in epicenter location have a negligible impact on the length of the inspected network. In contrast, hazard‐ and vulnerability‐related variables, including magnitude, ground‐motion median, soil class, and fragility medians, exert a strong influence. Increases in magnitude or ground‐motion median, or softer soils, markedly expand the network to be inspected, whereas lower component vulnerability, such as bridges and tunnels, reduces it. A variance‐based global SA extends the assessment to capture first‐order and interaction effects, supporting the prioritization of data collection and model refinement to achieve the greatest reductions in disruption. The methodology provides actionable guidance for infrastructure managers to target and enhance monitoring and mitigation strategies under bradyseismic crises.

## 1 Introduction

Sensitivity analysis (SA) is a cornerstone of engineering design and risk assessment, offering a systematic approach to dissect complex models and inform risk mitigation strategies. By quantifying how uncertainties in model inputs affect outputs, SA provides insights into the complex behavior of systems and guides decision‐makers in prioritizing resources (Pianosi et al. 2016). The influence of each variable is often assessed through its contribution to output variability, allowing analysts to identify dominant drivers of risk, screen out insignificant parameters, and better understand interactions between design and noise factors (Christopher Frey and Patil 2002). This makes SA especially relevant to seismic risk assessment (Burton 2023; Haukaas and Der Kiureghian 2005; Haukaas 2023; Tesfamariam et al. 2010), a field where reliable predictions are critical for ensuring safety, resilience, and cost‐effective planning (Padgett and DesRoches 2007; Dyanati et al. 2017; Iacoletti et al. 2024).

Seismic risk analysis typically integrates, among others, models of earthquake hazard, structural response, and system performance, each of which is affected by uncertainty (Foulser‐Piggott et al. 2020). These uncertainties are generally divided into aleatory, reflecting the inherent randomness of seismic events and considered irreducible, and epistemic, arising from limited knowledge or modeling assumptions and therefore potentially reducible (Bradley 2013; Sokolov and Wenzel 2019). A probabilistic framework, often based on the total probability law, combines hazard, vulnerability, and exposure to evaluate seismic risk (Taflanidis and Jia 2011; Cremen and Baker 2021). In practice, this framework is implemented through a sequential chain of modules, including hazard analysis, structural analysis (often via nonlinear dynamic time‐history simulations), damage analysis, and loss analysis, which progressively translate seismic actions into probabilistic performance measures and decision variables (Kourehpaz et al. 2025).

Traditional SA methods have relied heavily on local methods, such as one‐at‐a‐time (OAT) perturbations and partial derivatives (Conte et al. 2003; Gong et al. 2005; Roth and Grigoriu 2010). Known as local SA (LSA), these methods assess the effect of single parameters near central values and are often visualized with tornado diagrams (Cavalieri and Franchin 2020). Although LSA has been applied to evaluate the impact of alternative earthquake scenarios on losses (Neighbors et al. 2013), it neglects parameter interactions and explores only a narrow portion of the input space, which may lead to incomplete insights in problems characterized by strong dependencies (Haukaas and Der Kiureghian 2005).

To address these limitations, regional SA (RSA) and global SA (GSA) have been developed (Spear and Hornberger 1980; Saltelli et al. 2008). These methods examine the impact of uncertain parameters across various regions of the input–output space, making them well‐suited for spatially distributed infrastructure systems, such as transportation networks, utilities, or city‐scale portfolios (Abbadi and Lamdouar 2022). RSA helps identify thresholds or regions where changes in parameters trigger qualitatively different outcomes (e.g., exceeding damage limits or service disruption), thus supporting efficient preliminary screening and guiding data collection or model refinement (Chisari et al. 2018). GSA, particularly variance‐based methods such as Sobol's indices (Sobol 2001; Saltelli 2002), decomposes output variance into contributions from individual inputs and their interactions (Du and Wang 2024). Unlike local approaches, GSA accounts for the full range of input uncertainty, making it particularly powerful for seismic risk problems. Applications include probabilistic ranking of bridge retrofits in road networks (Bhattacharjee and Baker 2023), quantifying the benefits of repair interventions using seismic hazard (Ching et al. 2009), and studying how uncertainties in source parameters, ground‐motion models (GMMs), and fragility functions propagate to loss estimates (Vetter and Taflanidis 2012; Laporte et al. 2024). Case studies indicate that source parameters frequently dominate regional loss variability (Neighbors et al. 2013), whereas fragility models are particularly influential at the city scale (Cavalieri and Franchin 2020). The primary drawback of variance‐based GSA is its computational cost, as the reliable estimation of sensitivity indices may require thousands of model evaluations (Zhong et al. 2020; Liu et al. 2025).

Given the high computational demands of seismic risk models, which often involve nonlinear time‐history analyses, detailed damage models, or high‐dimensional finite element simulations, surrogate models (e.g., metamodels, emulators, and response surfaces) have become an essential complement. Methods, such as Gaussian process models, kriging, polynomial chaos expansions (PCE), ANOVA‐HDMR, and stochastic response surface methods (SRSM), provide efficient, accurate approximations of the original models, drastically reducing computational effort while enabling robust sensitivity and uncertainty analyses (Iooss and Le Gratiet 2019; Zentner and Borgonovo 2014; Zaicenco 2015). These surrogates have been applied to estimate fragility curves, optimize seismic mitigation devices, and support multi‐objective design problems (Zhong et al. 2020).

Regardless of the SA method, it has been demonstrated that integrating SA into decision‐support frameworks enhances risk‐informed infrastructure management. In the field of seismic risk, this includes quantifying the contribution of uncertain parameters to loss and downtime predictions (Kourehpaz et al. 2025) and managing risks in interdependent systems such as buildings, transportation, and lifelines (Burton 2023; Bhattacharjee and Baker 2023). By identifying the most sensitive variables, SA enables decision‐makers to target data collection, refine models, and design interventions with the most significant impact. Coupled with object‐oriented and modular workflows (Haukaas 2023) and ready‐to‐use toolboxes (Pianosi et al. 2015), SA provides a practical and adaptable toolset for infrastructure managers, policymakers, and engineers striving to enhance resilience in the face of seismic hazards.

In this study, SA is applied to the Campania railway network in Italy, currently exposed to bradyseismic activity (Cito et al. 2025). This is a real moment of crisis for both the local population and the government. Specifically, the sensitivity to the epicenter location, duration magnitude, GMM variability, soil type, and fragility curves for both railway masonry bridges and tunnels is studied. To keep the problem tractable, seismic risk is quantified as the length of railways requiring inspection after a seismic event. As inspection entails service interruption, this metric is used as a proxy for service disruption. This study is highly valuable to infrastructure managers, as it helps them understand which parameter is the most important to consider in decision‐making.

Following this introduction, the article is structured as follows: Section 2 presents the case study; Section 3 describes the methodology employed for the risk assessment; Section 4 discusses the results of the SA; and Section 5 provides the concluding remarks.

## 2 The Case Study

**FIGURE 1:**
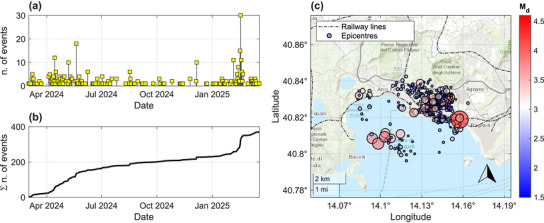
Bradyseism unrest: (a) number of events in 1 year, (b) time‐dependent seismicity, and (c) epicenter and magnitude distribution.

Campi Flegrei is a volcanic complex located on the western edge of Naples (Campania Region) in southern Italy. It encompasses several municipalities, with Pozzuoli as its central city (population approximately 75,000). The area is currently experiencing bradyseismic unrest, characterized by a slow, long‐term uplift of the caldera floor. This quasi‐circular, bell‐shaped deformation field extends approximately 10 km in diameter around Pozzuoli's historic center. Bradyseismic episodes are associated with intense seismicity (Figure 1a). Between April 2024 and April 2025, the area experienced approximately 400 earthquakes with a duration magnitude (*M*_d_) larger than 1.5 (Figure 1b). Most events cluster within the uplifted zone, consistent with their bradyseismic origin (Figure 1c).

In this study, duration magnitude is adopted because it is the first parameter released rapidly after the event by the Italian Institute of Geophysics and Volcanology (INGV). Its rapid availability makes it particularly suitable for emergency decision‐making.

**FIGURE 2:**
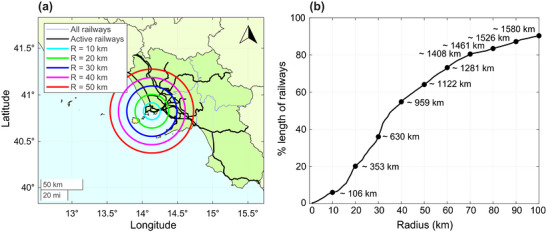
(a) Circles with increasing radius encompassing larger portions of railways; and (b) cumulative length of railways for increasing radius from a nominal epicenter in Pozzuoli.

For bradyseismic events with *M*_d_ ≥ 4, the leading national railway infrastructure operator in the area, *Rete Ferroviaria Italiana* (RFI), implements safety protocols based on the peak ground acceleration (PGA): Service suspension if the PGA is larger than 0.10 g; speed reduction to 30 km/h if PGA is included in the range between 0.05 and 0.10 g, and regular operation if PGA is lower than 0.05 g. These decisions are critical, given that the Campania railway network spans over 1750 km (Figure 2a). Specifically, the system comprises ∼30 lines (not all managed by RFI), with 22 currently active lines, serving a service range of 14 to ∼300 trains/day.

The railway geospatial data used in this study are sourced from OpenStreetMap, a comprehensive open‐source database for transport infrastructure (He et al. 2024).

Assuming a nominal epicenter in Pozzuoli (40.826° latitude and 14.122° longitude), the cumulative length of railways included within concentric circles of increasing radius can be computed. Such circles approximate an ideal distribution of ground‐motion intensity propagating radially from the epicenter (an elementary assumption). As shown in Figure 2b, the cumulative railway length grows sharply beyond a radius of 10 km and begins to increase more gently at approximately 40 km.

The length of railways requiring inspection following a seismic event is adopted as the outcome (a.k.a. decision variables) of the seismic risk analysis. This metric serves as a direct proxy for service disruption, as the required inspection length is proportional to both the total inspection duration and the consequent reduction in daily train service capacity. Importantly, the railway system also comprises several vulnerable components, such as railroads, tunnels, and bridges, that may experience significant seismic demand (Banimahd and Arbabi 2013; Argyroudis and Kaynia 2013). These elements are therefore crucial in determining the length of the railway to be inspected.

## 3 Scenario‐Based Seismic Risk Assessment

A seismic scenario is entirely defined by the epicenter location and magnitude, providing the basis for subsequent risk assessment. The scenario‐based seismic risk assessment formulation adopted in this study follows the approach outlined by De Risi et al. (2019). It consists of the convolution of a vulnerability and a hazard component (first and second terms within the integral in Equation 1):

(1) $$P\left(\mathrm{ls}|m,r\right)=\int_{\mathrm{IM}}P_{\mathrm{LS}|\mathrm{IM}}\left(\mathrm{ls}|\mathrm{im}\right)\cdot f_{\mathrm{IM}|M,R}\left(\mathrm{im}|m,r\right)\cdot d\mathrm{im}$$

where P(ls|m,r) represents the conditional probability of reaching or exceeding a specific limit state (ls) for a given seismic scenario, defined by magnitude *m* and epicentral distance *r*. This quantity can also be interpreted as the probability of failure. For clarity, the subscript *d* is dropped from the duration magnitude in the formulation. The epicentral distance *r* (usually expressed in km) is calculated as the distance between the epicenter and the infrastructure asset.

A limit state (ls) represents a discrete performance threshold that delineates acceptable from unacceptable system behavior, such as the onset of significant damage or near‐collapse conditions. The integration in Equation (1) is an application of the total probability law (Jaynes 2003).

**FIGURE 3:**
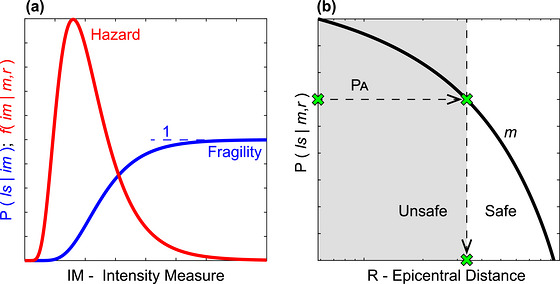
Schematic representation of the risk‐assessment methodology. (a) Fragility and hazard curves. (b) Safe distance identification.

The term $P_{\mathrm{LS}|\mathrm{IM}}\left(\mathrm{ls}|\mathrm{im}\right)$ in Equation (1) is the vulnerability component, illustrated as the blue curve in Figure 3a. It is a cumulative distribution function (CDF) that gives the conditional probability of reaching or exceeding a specific limit state for a given intensity measure (IM). In this study, the IM is defined as the PGA, which is the maximum absolute value of the acceleration recorded at the ground surface during an earthquake. Within performance‐based earthquake engineering (PBEE), the function $P_{\mathrm{LS}|\mathrm{IM}}\left(\mathrm{ls}|\mathrm{im}\right)$ is referred to as the fragility function (Porter et al. 2007) and is discussed further in Section 3.1.

The second term $f_{\mathrm{IM}|M,R}\left(\mathrm{im}|m,r\right)$ in Equation (1) is the hazard component, shown as the red curve in Figure 3a. It is the conditional probability density function (*pdf)* of the IM given the magnitude *m* and distance *r*, typically provided by empirical GMMs (Estacio and De Risi 2025). Details on the selected GMMs are provided in Section 3.2.

For a given magnitude *m*, varying the epicentral distance R allows the computation of a curve that maps R to the probability of failure, illustrated by the black curve in Figure 3b. By intersecting this curve with a tolerable probability of failure (*P*_A_), the critical epicentral distance can be obtained. Values below this threshold correspond to an unsafe system, whereas larger distances are deemed safe.

This epicentral distance can then be mapped onto the railway network to identify the length of railway infrastructure requiring inspection after a seismic event (Figure 2).

The selection of a tolerable probability of failure is inherently complex, lying at the intersection of ethics, economics, uncertainty, and engineering judgment. Nevertheless, design codes provide reference values for these parameters. Specifically, Eurocode 0 (BS (British Standard) 2002) establishes that for critical infrastructure, and considering an annual reference period, the acceptable probability of failure (*P*_A_) is 9.96 × 10^−8^. This value is adopted hereafter. However, any other values can be used here in other circumstances if deemed more appropriate; therefore, this assumption does not compromise the generality of the methodology.

### 3.1 Vulnerability Model

Railways are distributed interdependent lifelines (Galvan and Agarwal 2020) whose functionality depends as much on ground conditions as on structures. As tracks, tunnels, bridges, and earthworks operate effectively in series, the overall serviceability is governed by the weakest of these elements. Service disruption typically arises first from track‐geometry degradation (embankment instability, ballast fouling, and differential settlement). In contrast, discrete assets, such as bridges and tunnels, concentrate fragility at distinct locations (Miano et al. 2016; De Risi et al. 2017). Service disruptions are primarily driven by ground shaking and, in specific contexts, by permanent ground deformation (e.g., liquefaction, lateral spreading, landslides, and fault rupture). Additional vulnerability drivers include soil–structure interaction (Luo et al. 2025) and aging mechanisms (e.g., corrosion, fatigue; Pugliese et al. 2022; Grilli et al. 2025).

**FIGURE 4:**
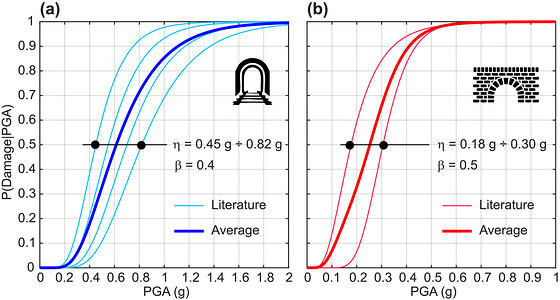
Fragility curves for (a) tunnels and (b) masonry bridges for moderate damage.

As outlined in Section 2, fragility‐based approaches are adopted here to quantify the conditional probability of damage for a given ground‐motion intensity, thereby enabling a direct mapping between seismic activity and network functionality. Figure 4 shows the fragility curves and associated variability used in this study. Consistent with the characteristics of bradyseismic events, only the limited‐damage limit state is considered, as collapse is unlikely under such shaking. Significantly, even limited damage may still disrupt railway operations, for instance, due to bridge deformations or the risk of debris obstruction on the tracks within a tunnel.

Tunnels perform well under pure shaking when they are deep in rock, because confinement limits distortion. By contrast, vulnerability increases in shallow cut‐and‐cover segments, particularly at portals and transition zones, where strains tend to concentrate. In the bradyseismic context, the focus is restricted to ground shaking, with permanent ground deformation being neglected due to the physical nature of the phenomenon. According to ALA (American Lifelines Alliance) (2001), bored circular tunnels in rock have lower probabilities of moderate to extensive damage at a given shaking level than soft‐ground cut‐and‐cover boxes. Fragility also depends on the lining type, joint spacing, flexibility, and cover‐to‐diameter ratio. Figure 4a shows the four ALA's fragilities adopted herein, corresponding to different tunnel typologies at the moderate damage limit state, which forms the basis of the vulnerability variability hereafter.

Masonry arch bridges, the predominant typology in the network, are susceptible to local (e.g., spandrel wall overturning) and global failure mechanisms (e.g., transverse response of piers in multi‐span systems), with serviceability often governed by shear/flexural checks of substructures (Manzini et al. 2021). Figure 4b presents fragility functions for single‐span and multiple‐span arch bridges, derived from the literature (Manzini et al. 2021). In the present case study, high‐speed lines are excluded from the vulnerability assessment because they do not include masonry arch bridges.

The fragility for both bridges and tunnels is modeled with a lognormal CDF:

(2) $$P_{\mathrm{LS}|\mathrm{IM}}\;\left(\mathrm{ls}|\mathrm{im}\right)=\Phi \left(\frac{\log\mathrm{im}-\log\eta }{\beta }\right)$$

where η is the median, and β is the logarithmic standard deviation. For the tunnels, the median of the lognormal model ranges from 0.45 to 0.82 g, and the logarithmic standard deviation is fixed at 0.4. For bridges, the median varies between 0.18 and 0.30 g, and the logarithmic standard deviation is fixed to 0.5.

### 3.2 Hazard Model

GMMs, also known as ground‐motion prediction equations (GMPEs), are the primary tool for estimating surface shaking intensity from source parameters. Their application to volcanic seismicity, however, presents unique challenges due to distinct source mechanisms and attenuation characteristics. Volcanic seismicity arises from processes, such as magma migration, hydrothermal activity, and pressure changes within calderas, as well as brittle faulting. This results in shallow, often moderate‐magnitude earthquakes that, due to their proximity to the surface and infrastructure, can produce intense local shaking (Lanzano and Luzi 2020).

Conventional GMMs calibrated on tectonic events often misestimate shaking in volcanic areas due to the differences in attenuation characteristics and frequency contents. For example, studies at Mount Etna and Ischia (both in Italy) have shown that shallow volcanic earthquakes attenuate more rapidly with distance and contain higher low‐frequency energy than deeper tectonic events (Tusa and Langer 2016). Consequently, volcano‐specific GMMs that account for local geology and source conditions are essential for reliable hazard assessment. Recently, new volcanic‐specific GMMs have been developed for the Campi Flegrei area (Scala et al. 2026). Historically, seismicity in this area has been reported using duration magnitude (*M*_d_), derived from signal coda length. However, GMMs typically employ moment magnitude (*M*_w_), which is physically tied to seismic moment and energy release. The distinction is important as follows: *M*_w_ provides a consistent measure across regions and magnitudes, whereas *M*_d_ can saturate at larger magnitudes but is more computationally efficient in real‐time applications. A recent study (Scala et al. 2026) has established spectral inversion‐based conversions between *M*_d_ and *M*_w_, enabling robust GMM calibration. From an operational standpoint, *M*_d_ retains advantages, as it can be estimated quickly and robustly from local networks during seismic swarms, providing a real‐time proxy for earthquake size before spectral analyses are completed. In practice, *M*_w_ governs long‐term hazard models, whereas *M*_d_ enhances rapid situational awareness, creating a complementary framework for both planning and emergency management.

**FIGURE 5:**
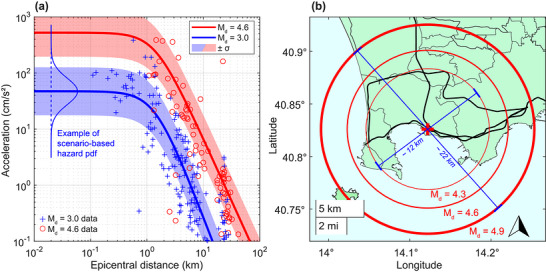
(a) Examples of GMMs and (b) 10 cm/s^2^ iso‐acceleration contours for magnitude duration 4.6 ± 0.3.

The functional form of the GMM used herein is that proposed by Scala et al. (2026):

(3) $$\mathrm{lo}g_{10}\mathrm{IM}\;∼\;N\left[a+bM+\left(c+c_{2}M\right)\cdot \mathrm{lo}g_{10}\sqrt{R^{2}+h^{2}}+eS;\sigma \right]$$

where the coefficients *a*, *b*, *c*, *c_2_*, *h*, and *e* are provided in Scala et al. (2026) for the PGA. The logarithmic standard deviation *σ* is also provided. Figure 5a illustrates the GMM behavior with respect to magnitude and epicentral distance, with markers indicating the recorded ground motions in Campi Flegrei by the local seismic network.

In Equation (3), *M*, *R*, and *S* are the duration magnitude, the epicentral distance, and the soil term, respectively. *S* is a dummy variable equal to 0 for an assessment on rock and 1 for an evaluation on softer soils. According to Equation (3), the hazard component in Equation (1) is log‐normal; Figure 5a shows an example of the hazard *pdf* $f_{\mathrm{IM}|M,R}\left(\mathrm{im}|m,r\right)$. The logarithmic standard deviation σ represents the aleatory variability of the model; however, it is essential to note that the central estimate of the GMMs can be uncertain itself. In fact, the model coefficients (i.e., *a*, and *b*) are also provided with percentiles, allowing for the propagation of epistemic uncertainty.

Other possible effects leading to variability in the hazard include the epicenter location and the estimated value of the duration magnitude, reflecting variability due to data quality and inversion processes. For a site with a dense accelerometer network, the error in the moment duration can range from ±0.1 to ±0.3. Figure 5b shows the effect of such variability by plotting the iso‐acceleration contours corresponding to 10 cm/s^2^. For a magnitude of 4.6, the maximum recorded in Campi Flegrei, the covered area increases from 12 to 22 km, considering a magnitude variability of ±0.3.

## 4 Sensitivity Analysis

Six epistemic uncertainties are propagated through the scenario‐based seismic risk assessment as follows: (1) epicenter location error, *δ*_e_, (2) azimuth error, *ϕ*, (3) error in estimated duration magnitude, *δ_M_*_d_, (4) uncertainty in the central value of the GMM, *δ*_GMM_, (5) variability of the soil term in the GMM, *S*, and (6) uncertainty in the median value of the fragility curves, *η*. Table 1 lists the assumed distributions (and their parameters) for each of the uncertain variables mentioned above.

**TABLE 1:** Input parameters for the sensitivity analysis (*μ* and *σ* are the mean and standard deviation).

	Variable	Distribution	Parameters
*δ*_e_	Epicenter	Uniform	min = 0 km; max = 0.5 km
*ϕ*	Azimuth	Uniform	min = 0°; max = 360°
*δ_M_*_d_	Magnitude	Uniform	min = −0.2; max = +0.2
*δ*_GMM_	Median GMM	Uniform	min = 0.5; max = 2.0
*S*	Soil	Discrete Uniform	min = 0; max = 1
*η*	Median fragility	Uniform	Tunnels: min = 0.45; max = 0.82
			Bridges: min = 0.18; max = 0.30

The first two parameters capture epicenter‐related uncertainties. As mentioned earlier, a nominal epicenter is considered (i.e., 40.826° latitude and 14.122° longitude), with errors reflecting the performance of the seismic network. Fuggi et al. (2024) report uncertainties under 200 m within well‐instrumented areas and over 600 m at network edges, suggesting an average epicenter‐location error approaching 500 m across the domain. This uncertainty is modeled as a uniform distribution with extremes 0 and 500 m from the epicenter. In this application, the distance is not enough to identify the new location of the epicenter; there is also a need to assess the direction along which the distance error is computed. Therefore, an azimuth angle is introduced as an additional uncertainty. The addition of the azimuth in the SA is meaningful, as it indicates the potential geographic area in which an earthquake can be more impactful if recognized as an influential parameter. The third random variable reflects possible errors in magnitude assessment due to seismic recording quality. Although duration magnitude errors rarely exceed ±0.3 (Tsumura 1967), in dense networks like Campania's, errors can be as small as ±0.1. Accordingly, a conservative ±0.2 is adopted here (reported in Table 1).

The fourth parameter represents epistemic uncertainty in GMM. GMMs in volcanic settings are inherently less constrained than tectonic analogues (Foulser‐Piggott 2014); thus, a variability range from half to double the predicted value is used. The parameter about the soil evaluates the importance of site conditions. According to the GMM formulation in Equation (3), *S* is a variable that can be either 0 or 1. Finally, the sixth random variable captures the limited knowledge regarding infrastructure vulnerability, as outlined in Section 3.1. Below, the results of an RSA are presented, followed by more rigorous GSA outcomes that are discussed. RSA and GSA are carried out using the SAFE toolbox (Pianosi et al. 2015).

The following section presents a SA conducted using a simulation‐based approach. To ensure the repeatability of the SA (Iman and Helton 1991), an adequate number of simulations are conducted for both the RSA and GSA. Moreover, it is essential to emphasize that the risk model presented in Section 3 is not stochastic, that is, it returns the exact value of the length of railways at risk for the same inputs. This is an additional aspect that ensures repeatability for such a complex risk assessment.

### 4.1 Regional SA

**FIGURE 6:**
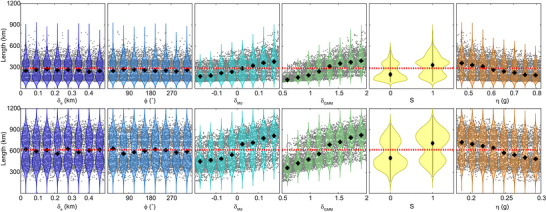
Scatter plots correlating the length of at‐risk railways versus epicenter, azimuth, magnitude, GMM, soil, and vulnerability variabilities for the case of tunnels (first row) and bridges (second row) and *M*_d_ = 4.6.

Figure 6 shows scatter plots of at‐risk railway length against the six uncertain variables, based on 6000 samples (*N* = 1000 × number of variables) generated via Latin‐hypercube sampling, ensuring stable visual patterns in the scatterplots and stable empirical distributions. Increasing *N* beyond 1000 did not materially change the correlation structure or the RSA conclusions. Both tunnels and masonry bridges under *M*_d_ = 4.6 (the maximum recorded magnitude at Campi Flegrei) are shown. Results for lower magnitudes are consistent. The scatter plots are represented with violin plots, which show the variability and the median computed for a predefined number of bins.

The RSA highlights (1) no observational correlation between risk and epicenter uncertainties (i.e., *δ*_e_ and *ϕ*), (2) a mild positive correlation with hazard‐related parameters (i.e., *δ_M_*_d_, *δ*_GMM_, and *S*), and (3) a negative correlation with vulnerability parameters (as stronger structures reduce risk). Similar patterns are observed for tunnels, though the correlations are higher because they generally have higher seismic capacity than masonry bridges.

**FIGURE 7:**
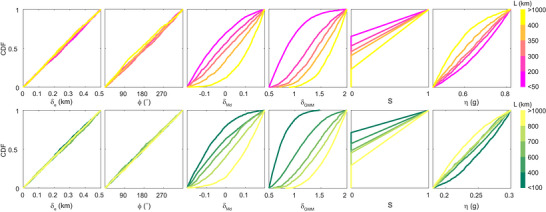
Empirical CDFs of input factors associated with output values within five different ranges of at‐risk length of railway for the epicenter, azimuth, magnitude, GMM, soil, and vulnerability variabilities for the case of tunnels (first row) and bridges (second row) and *M*_d_ = 4.6. CDFs, cumulative distribution functions.

Figure 7 shows the empirical CDFs of the input factors across 5 different ranges of the at‐risk railway length. Results for *δ*_e_ and *ϕ* confirm the lack of correlation between the risk metric and the epicenter's variabilities. Results for the hazard components (i.e., *δ_M_*_d_, *δ*_GMM_, and *S*) reveal that higher magnitudes, larger GMM scaling, and softer soils dramatically increase the length of the at‐risk railway. Finally, the CDF of the vulnerability component (*η*) confirms that greater fragility curve medians (i.e., stronger assets) reduce risk.

This preliminary RSA indicates that hazard parameters have a significant influence on the risk metric, whereas epicenter errors are negligible.

### 4.2 Identification of Output Clusters

Before conducting the variance‐based SA, a preliminary check is performed on the data to ensure that distributional features do not unduly influence the results. Specifically, multimodality is assessed using the Hartigan dip test (Hartigan and Hartigan 1985), and skewness is evaluated through the D'Agostino test (D'Agostino 1970). Although these diagnostic steps are not strictly required for carrying out the SA, they provide helpful reassurance about the underlying statistical properties of the inputs. By including these tests as a sanity check, it is ensured that the subsequent SA is not biased or distorted by hidden asymmetry or irregularities in the data distribution. The statistical tests mentioned above indicate that both tunnels and bridges are skewed and multimodal. These findings are consistent with what can be observed in Figure 6, where a clear separation is evident between two distinct groups of samples. Figure 6 indeed shows that the risk results tend to cluster in two main groups. A sharp separation is visible both in the cluster plots and in the shape of the violin plots. A simple *k*‐means clustering algorithm (Lloyd 1982) identifies sharp separations in the at‐risk length at approximately 287 km for tunnels and 617 km for bridges (the red dashed lines in Figure 6). Therefore, the apparent gaps in the results arise from the bimodal clustering discussed here and do not represent a sampling artifact. The following results should be interpreted with these observations in mind.

### 4.3 Global SA

A variance‐based GSA is performed using the pick‐and‐freeze method (Saltelli et al. 2010), requiring *N* × (*k* + 2) model evaluations, where *k* = 6 is the number of input variables. A base sample size of *N* = 4500 is used, yielding 36,000 simulations, which ensures narrow bootstrap confidence intervals for the sensitivity indices (Pianosi et al. 2016). Following Saltelli et al. (2010), both first‐order and total‐order indices are computed, which represent the main and total effects for the variance‐based sensitivity measures. First‐order (or main effects) Sobol’ indices ($S_{i}$) quantify the fraction of output variance reducible by fixing a single input. Total‐order indices ($S_{T_{i}}$) measure the total contribution, including all interactions, indicating the variance that would remain if that input alone were uncertain. A large gap between $S_{i}$ and $S_{T_{i}}$ indicates significant interaction effects. The main and total indices are provided in Equations (4) and (5), respectively, and are estimated using the Saltelli–Jansen method (Saltelli et al. 2010):

(4) $$S_{i}=\frac{\mathrm{Va}r_{X_{i}}\left(E_{∼X_{i}}\left[L|X_{i}\right]\right)}{\mathrm{Var}\left(L\right)}$$

(5) $$S_{T_{i}}=\frac{E_{∼X_{i}}\left[\mathrm{Va}r_{X_{i}}\left(L|X_{∼i}\right)\right]}{\mathrm{Var}\left(L\right)}=1-\frac{\mathrm{Va}r_{X{∼}_{i}}\left(E_{∼X_{i}}\left[L|X_{∼i}\right]\right)}{\mathrm{Var}\left(L\right)}$$

where Var and E are the operator variance and expected value, respectively, L and $X_{i}$ are the length of the railway at risk and the parameters of the model, and the notation $X_{∼i}$ indicates the set of parameters except $X_{i}$.

**FIGURE 8:**
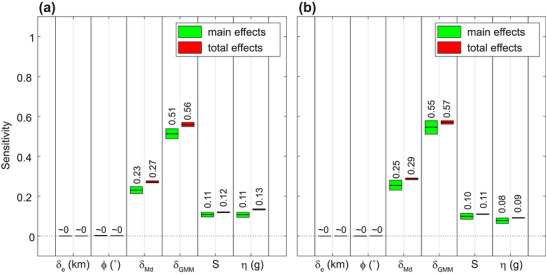
Main and total effects for (a) tunnels and (b) bridges for *M*_d_ = 4.6.

Figure 8 reports the main and total effects for the six examined parameters. The boxes represent the 95% bootstrap confidence intervals obtained considering 2000 extractions, whereas the central black lines indicate the bootstrap mean. Examination of the main effects shows that the epistemic variability in the central estimate of the GMM is the most influential parameter, followed by the magnitude. The third and fourth most influential parameters are the soil characterization and the median of the fragility curve, respectively. As expected from the RSA, the parameters on the error estimation of the epicenter location are uninfluential. Also, total‐effect indices exceed their corresponding main‐effect indices. In this case, this difference is minimal, denoting the model as additive. Notably, the bootstrap confidence intervals are quite small, confirming that the number of simulations is adequate (Sarrazin et al. 2016).

The calculation of total‐order Sobol indices, although computationally more expensive, is necessary at least once because the additive or nonadditive character of the model is not known a priori. Additivity can only be assessed after observing whether total‐effect indices significantly differ from first‐order ones. In this application, the small gaps between $S_{i}$ and $S_{T_{i}}$ for all parameters indicate that the risk model is largely additive within the considered uncertainty ranges. However, this conclusion could not have been established without first computing the total effects. Furthermore, the presence of a binary variable (*S*) and multiplicative scaling (*δ*_GMM_ × PGA) justifies checking for interactions, because nonlinear effects cannot be excluded a priori.

### 4.4 GSA for the Two Clusters

To investigate whether parameter sensitivity differs between the two distinct risk regimes (clusters) identified in Section 4.2, a second GSA (outlined in Section 4.3) is conducted. Given the computational cost of repeating a full Monte‐Carlo‐based GSA for each cluster, an efficient PCE surrogate model is employed (Xiu and Karniadakis 2002; Sudret 2008). The latter provides an efficient nonintrusive approximation of the risk model, enabling Sobol's indices to be computed analytically once the surrogate is trained. The following describes the formulation, calibration, and validation of the PCE models constructed for tunnels and bridge assets, which form the basis for the cluster‐specific SA.

Let Y=f(X) denotes the scenario‐based seismic risk model described in Section 3, where the input vector X comprises the six epistemic variables listed in Table 1. A PCE surrogate provides an exact functional approximation $\hat{Y}$ of the model response as a finite sum of orthogonal polynomials, as shown in the following equation:

(6) $$Y\approx \hat{Y}=\sum_{k=0}^{P}c_{k}\;\Psi_{k}\left(X\right)$$

where $\Psi_{k}$ is multivariate orthogonal basis functions, and $c_{k}$ is the corresponding expansion coefficients. The index 𝑘 enumerates all retained polynomials in the truncated expansion, ordered by increasing total degree and interaction level. Each basis function is constructed from univariate polynomials that are orthogonal with respect to the marginal distribution of each input variable, following standard PCE theory (Xiu and Karniadakis 2002; Sudret 2008). The multivariate basis is generated by tensorization of these univariate families, enabling the surrogate to represent the combined effect of all input variables on the model output.

The PCE can be constructed using a hyperbolic truncation scheme, which restricts the maximum polynomial degree while controlling the order of interactions among input variables. The admissible multivariate basis functions satisfy in the following equation:

(7) $$∥\alpha {∥}_{q}={\left(\sum_{i=1}^{d}\alpha_{i}^{\;q}\right)}^{1/q}\leq p$$

where α is the multi‐index defining the polynomial degree in each dimension, q<1 attenuates high‐order interaction terms and promotes sparsity, and p specifies the overall expansion order. This truncation strategy is widely used in sparse PCE to balance accuracy and computational efficiency (Blatman and Sudret 2010; Pinto et al. 2025). To further reduce dimensionality, the polynomial basis was refined using Least Angle Regression (LAR), which iteratively selects only the most informative terms. Following the adaptive sparse PCE framework of Blatman and Sudret (2011), this procedure yields a sparse expansion that preserves predictive accuracy while limiting overfitting and unnecessary high‐order terms.

A subset of input–output pairs was extracted from the simulation performed in Section 4.3, ensuring consistency between the simulation‐based GSA and the surrogate modeling stage. The PCE coefficients were then estimated through regression, using the LAR to obtain a sparse expansion that retains only the most informative basis functions (Blatman and Sudret 2011). The accuracy of the PCE surrogates was assessed using several complementary error metrics. The leave‐one‐out cross‐validation error $\varepsilon_{\mathrm{Loo}}$, was computed analytically from the PCE influence matrix. The relative generalization error was then estimated using the validation subset, providing a measure of $L^{2}$ prediction error relative to output variance. Across all cases, the final surrogates achieved $R^{2}>0.98$ with no noticeable degradation near the cluster thresholds, confirming that they accurately reproduce the behavior of the full deterministic risk model and are suitable for computing Sobol's indices.

**FIGURE 9:**
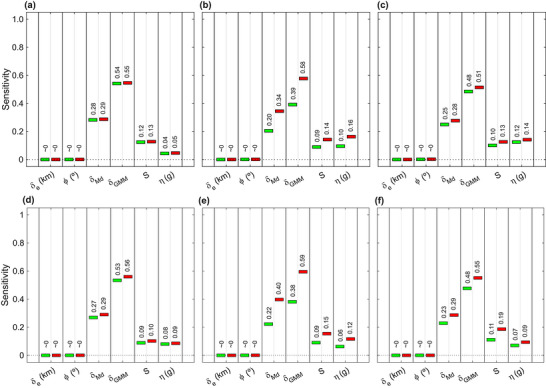
Main and total Sobol’ effects for tunnels and bridges across risk clusters by means PCE (a) Tunnels—full dataset (no clustering). (b) Tunnels—lower length cluster (<287 km). (c) Tunnels—upper length cluster (≥287 km). (d) Bridges—full dataset (no clustering). (e) Bridges—lower length cluster (<617 km). (f) Bridges—upper length cluster (≥617 km).

Figure 9 presents the main and total effects obtained with the PCE for tunnels (a–c) and bridges (d–f), considering the full dataset and the two output clusters. Figure 9a (for tunnel) and Figure 9d (for bridge) show the results using the full dataset (i.e., without applying the identified clusters), whereas Figure 9b and 9c (for tunnel) and Figure 9e and 9f (for bridges) display the lower and upper clusters defined in Section 4.2. The main and total effects using the full dataset for each asset closely match the Saltelli–Jansen estimates from Section 4.3 (comparing Figure 8a with Figure 9a and Figure 8b with Figure 9d), confirming the accuracy of the PCE surrogate and the consistency of the global sensitivity pattern. This agreement supports the use of PCE‐based variance decomposition for the subsequent cluster‐specific analysis.

For the lower length tunnel cluster (length <287 km), Figure 9b indicates that the ranking of influential parameters is consistent with the full‐dataset results. The main distinction in this cluster is the marked increase in higher order effects, reflected in the larger gap between main and total contributions. These increases arise primarily from interactions involving GMM scaling, magnitude error, soil class, and the median of the fragility curve with other parameters. This indicates that for modest shaking levels, the inspected length becomes more sensitive to joint variability in the hazard parameters. In the upper length tunnel cluster (Figure 9c), the hierarchy of main contributions is preserved, and the model response behaves in a more additive manner, similar to the full‐dataset analysis. This pattern indicates that, in high‐impact regimes, the response is governed mainly by the direct effect of the leading hazard terms.

Figure 9e shows that the lower length bridge cluster (length <617 km) follows a sensitivity pattern broadly aligned with the tunnel cluster and the full dataset. As in tunnels, this cluster displays higher interaction effects compared to the full‐dataset results. The most influential interactions again involve the GMM scaling factor and the magnitude error, indicating that joint variability in hazard parameters becomes more relevant under moderate shaking levels. In contrast, Figure 9f shows that the upper length bridge cluster retains the same types of higher order effects observed in the lower length group, but with substantially reduced interaction levels. This suggests that, for bridges, compound variability in the hazard field remains relevant even under more severe shaking.

**FIGURE 10:**
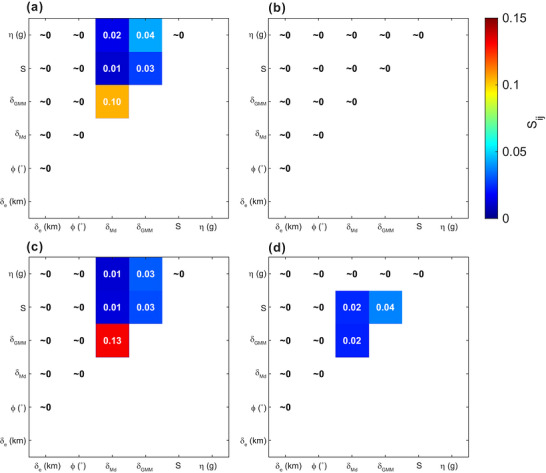
Second‐order Sobol’ effects for tunnels and bridges across risk clusters. (a) Tunnels—lower length cluster (<287 km). (b) Tunnels—upper length cluster (≥287 km). (c) Bridges—lower length cluster (<617 km). (d) Bridges—upper length cluster (≥617 km).

Figure 10 summarizes the higher order Sobol's contributions (Equation 8) for both asset types, showing that secondary effects arise almost exclusively from second‐order interactions:

(8) $$S_{ij}=\frac{\mathrm{Var}\left(E\left[L|X_{i},X_{j}\right]\right)-\mathrm{Var}\left(E\left[L|X_{i}\right]\right)-\mathrm{Var}\left(E\left[L|X_{j}\right]\right)}{\mathrm{Var}\left(L\right)}$$

For tunnels (Figure 10a,b), interactions are concentrated in the lower risk cluster, where the most relevant terms involve GMM scaling with magnitude error, and the median of the fragility curve with the GMM scaling factor. These contributions, although moderate, are notably larger than those obtained using the full dataset, indicating that joint variability among hazard‐related parameters becomes more influential when shaking levels remain within the lower range of affected length. In contrast, second‐order effects for the upper length tunnel cluster are minimal, confirming the nearly additive behavior observed in the main‐ and total‐effect analysis.

For bridges (Figure 10c,d), interaction patterns differ substantially. In the lower length cluster, second‐order effects are driven primarily by the same hazard‐related combinations observed for tunnels. However, in the upper length cluster, interaction terms increase modestly, particularly those involving soil class, GMM scaling, and magnitude error. This amplification reflects the lower median capacities of masonry arch bridges, which make the inspected length more sensitive to compound variations in both hazard and vulnerability parameters when shaking reaches higher intensities.

In conclusion, although the full‐dataset GSA indicates that the model behaves nearly additively, the cluster‐specific PCE analysis reveals that non‐negligible interaction effects emerge in the lower impact regime, particularly involving GMM scaling, magnitude error, and fragility medians. It is also worth noting that, unlike tunnels—which become almost fully additive in the upper length cluster—masonry bridges retain modest but persistent second‐order interaction effects even in high‐impact conditions, reflecting their lower median capacities.

## 5 Conclusions

This study assessed the seismic risk of the Campania railway network during the ongoing bradyseismic crisis at Campi Flegrei using both regional and global sensitivity analyses. Risk was framed in terms of the railway length requiring inspection after a seismic event, providing a practical operational proxy for service disruption. The results showed that uncertainties in epicenter location are negligible. In contrast, hazard‐related parameters (magnitude, ground‐motion median, and soil type), together with fragility medians, exert the most decisive influence on risk outcomes. GSA further highlighted the dominant role of hazard‐related variability, underscoring the value of seismological and geotechnical characterization and refined hazard modeling. Overall, the findings demonstrate that SA offers a powerful means of guiding infrastructure managers by pinpointing the most critical sources of epistemic uncertainty. In practice, this enables more targeted data collection, monitoring, and model refinement, ultimately supporting robust, risk‐informed decision‐making during volcanic seismic unrest. However, it should be noted that as some epistemic uncertainties—such as GMM central‐value variability and moment‐duration estimation—are governed by external seismological agencies rather than the infrastructure manager, their high sensitivity does not directly translate into actionable mitigation within the railway authority's domain.

Specifically, the SA offers a clear, prioritized roadmap for the railway infrastructure manager to maximize risk‐reduction effectiveness per unit of investment. The primary conclusion is that efforts to reduce the epistemic uncertainty of the input parameters will yield far greater returns than attempting to address the inherent variability (aleatory uncertainty) of the model output. In particular, the infrastructure manager should focus on the most influential variables within their direct sphere of action, such as soil class and fragility parameters. Targeted structural assessments should be carried out to develop asset‐specific fragility functions for the most vulnerable structures. Immediate actions should also include investing in high‐resolution geotechnical surveys along critical railway segments to precisely define the binary stiff/soft boundary, thereby reducing a major controllable source of uncertainty in the model.

Conversely, parameters related to the volcanic GMM and the magnitude estimation largely depend on external institutions (e.g., the national geological service) and therefore lie outside the direct control of the infrastructure manager. Although ongoing improvements by these authorities, such as densifying the accelerometric network or enhancing real‐time magnitude estimation procedures, will benefit the overall hazard characterization, they should not be considered primary investment targets for the infrastructure manager, unless joint efforts are established. Likewise, further efforts to improve the precision of epicentral location are not cost‐effective from the perspective of railway‐specific risk, as the SA showed that this uncertainty has a negligible influence on the at‐risk length of the network.

Looking forward, future research should extend the framework to incorporate vertical acceleration and cascading hazards, such as ground deformation, liquefaction, and landslides, which may interact with seismic effects in volcanic regions. Advances in surrogate modeling and adaptive sampling could reduce computational demands and enable broader exploration of uncertainty spaces without compromising accuracy. Expanding the scope beyond tunnels and bridges to include additional railway components, supported by improved fragility models, would further enhance predictive capability. Finally, integrating the methodology with real‐time monitoring and decision‐support tools would strengthen operational resilience, enabling infrastructure managers to adapt dynamically during bradyseismic crises while also informing long‐term regional planning.

## Funding

This study was supported by Economic and Social Research Council (ES/Z000122/1).

## Data Availability

The data that support the findings of this study are available on request from the corresponding author. The data are not publicly available due to privacy or ethical restrictions.
